# **A study of the impact of social network types on the health of older adults**—**Mediated by psychological resilience**

**DOI:** 10.1371/journal.pone.0333966

**Published:** 2025-10-16

**Authors:** Yuan Chai, Qi Zhang, Yifei Yuan

**Affiliations:** School of Labor Economics, The Capital University of Economics and Business, Beijing, China; Hong Kong Shue Yan University, HONG KONG

## Abstract

**Aim:**

To explore the different types of social network types and their impact on the health of the elderly, as well as the influencing mechanisms to promote their health.

**Method:**

k-means clustering, regression analysis, mediating effect method.

**Results:**

For Chinese elderly people, social network types have a significant impact on the self-rated health, but not on the chronic conditions. The benefits of diverse social network types are significant and positively influence their health by enhancing psychological resilience. There is heterogeneity among different characteristic groups.

**Discussion:**

This not only suggests that elderly individuals should consciously engage with different types of people in their daily lives, but also emphasizes the need for the government to further establish and improve platforms that promote a diverse social network for the elderly, and provide personalized services and guidance for different groups.

## 1. Introduction

**The increasingly large elderly population also means that the health of the elderly requires more attention and discussion.** Population aging has become a global demographic phenomenon, with the number of people aged 60 years or over expected to reach 2.1 billion by 2050 [1]. Due to declining physical functions, older adults are more likely to develop various chronic diseases, which significantly affects their quality of life and health outcomes, posing major challenges to global healthcare systems and social service systems. In developed countries, population aging has led to increased healthcare expenditures and pressure on pension systems [2]. Similarly, for China, according to the National Health Commission in July 2019, more than 180 million elderly people in China are suffering from chronic diseases, with the proportion reaching 75%. “Longevity without health” will not only greatly affect the quality of life of the elderly themselves, but will also have a huge impact on China’s social security, healthcare services and social services, etc [3]. Therefore, addressing the health challenges of the elderly in the context of population aging has become a critical policy priority requiring immediate attention and systematic solutions.

Health is a multidimensional concept encompassing physiological, psychological, social adaptation, and moral aspects. Due to this multidimensionality, there are numerous health measurement indicators. Indicators measuring objective health mainly include mortality rates, morbidity rates, and ability to perform daily activities [4,5,6]. Indicators measuring subjective health primarily use self-rated health [7,8,9]. Notably, cardiovascular diseases, cancers, chronic respiratory diseases, and diabetes constitute the main health burden among the elderly population [2]. Self-rated health is a comprehensive indicator reflecting population health; it represents research subjects’ overall subjective perception of their objective health status and can effectively measure respondents’ health [10].

**The health status of older adults is influenced by multiple factors, with social networks playing an important role [11]. Social network is social relationship maintained by an individual that provides resources and support and is an important contributor to health.** The World Health Organization considers biology, environment, lifestyle, and health services as the four major factors that influence health. Social networks have received attention from scholars as part of lifestyle. The expansion and good quality of social networks can play a positive role in alleviating loneliness, effectively lowering the level of depression, and, improving life satisfaction among older adults [12,11].

**Social network pattern is a new perspective to study social networks, and how it affects the health of the elderly and what is the mechanism of influence need to be explored continuously.** Social network pattern refers to a categorization of social networks based on the integration of main features including network scale, composition, and functional support. It reflects the size of social networks and presents the distribution of composition and interaction status within these networks [13]. This classification approach enables researchers to capture the complexity of interpersonal environments that elderly individuals navigate in their daily lives. While network patterns across countries share similarities, they also reflect unique national characteristics. Scholars in Israel, the United States, and South Korea have classified social network types based on the number of members in each type of social network, the share of the total number of relationships, the frequency of interactions, and the proportion of support provided by different kinds of relationships [14,15,16]. Among them, Litwin’s proposed diverse, family, friend, and limited types are widely recognized by the academic community [14].

**In studies examining the impact of social network patterns on health, different social network patterns have varying effects on health.** People with diverse social network patterns tend to have better health outcomes, while those with limited patterns show the poorest outcomes [17,18,19]. Perhaps due to cultural differences, scholars disagree about the relative importance of family-based versus friend-based social network patterns on health. Western scholars believe friend-based social network patterns have a greater impact [15], while Eastern scholars generally argue that family-based social network patterns are more influential [16,9]. Some researchers have found no significant difference between the two types [19].

**Current research on the impact of social network patterns on health in China is limited, with data coming only from specific regions.** Among the few existing studies, some researchers have used survey data to study elderly health conditions in Chongqing and Sichuan [19], while others have conducted health surveys of elderly people in Jiayu County, Hubei [9]. There is a need for research based on national data.

In conclusion, social network patterns, which better reflect the complexity of interpersonal environments [13], have become a popular research topic in recent years. However, research on elderly health remains limited and largely focuses on direct effects, with mechanisms of influence requiring further analysis.

To explore the pathways through which social network patterns affect elderly health, this paper reviews relevant theories.

The convoy model suggests that an individual’s social relationship network provides emotional and instrumental support to the person who occupies the central position at different life stages, protecting their physical and mental health. In this model, these social relationship networks are called convoys [20]. Looking at the actual situation of elderly people, firstly, the elderly are surrounded by many convoy members who together form a complete social network. Secondly, the multifunctional support from convoy members in terms of economic and emotional aspects provides important assistance in improving the quality of life for the elderly. Specifically, in terms of social network pattern classification: government, society, community, and family jointly ensure the quality of life in old age. For example: the government provides basic pension, medical insurance, and quality public services for the elderly; communities provide activity venues, elderly care service facilities, and smart elderly care services; families provide economic support, daily care, and emotional support for the elderly.

**From the convoy model, we can see that all convoy members serve as strong support for the elderly, and such convoy support actually enhances the elderly’s ability to cope with various situations in aging.** The elderly can receive emotional and instrumental support from their caregivers, satisfying their need for intimacy and fulfillment through interaction. This helps them resist the sense of maladjustment caused by aging-related decline in economic resources, cognitive abilities, and physical health [21], as well as the impact of random negative events, thereby ensuring their physical and mental health and quality of life in old age.

This draws our attention to psychological resilience. Psychological resilience refers to a person’s ability to adapt and recover when facing a series of risks [22]. The academic community generally believes that psychological resilience is an important component of positive psychology [23,24]. Moreover, with population aging, psychological resilience has also been introduced into research on elderly populations [25], particularly in studies examining the concept of resilience in the elderly and its role in successful aging.

Regarding psychological resilience, there is currently no direct research on the relationship between social network patterns and psychological resilience. Therefore, by examining the relationship between social networks and psychological resilience, it is found that strong social networks can enhance psychological resilience. Utilizing internal and external resources is one of the ways to improve psychological resilience, with family, friends, and society being the main providers of external resources. Forming intimate relationships and participating in social activities are beneficial for improving individual psychological resilience [26]. Social support has a direct impact on psychological resilience [27,28].

Regarding the relationship between psychological resilience and health, good psychological resilience enables individuals to maintain positive and optimistic emotions when facing stress and difficulties, seek positive coping methods, accept themselves more openly, find joy amid hardship, and reduce the incidence of depression [29], thereby reducing the physical harm caused by negative emotions such as anxiety and depression [30]. Research based on Swedish military recruitment cohorts found that psychological resilience is negatively correlated with the incidence of early heart failure [31]. When elderly people encounter common and special difficulties such as declining physical function, cognitive ability, and socioeconomic status, psychological resilience can enhance individual self-regulation ability, promote active cooperation with treatment and rehabilitation [32], promote positive physical functioning [33], and serve a protective role, thus achieving physical and mental health [34].

It can be seen that existing literature has confirmed the positive effect of social support on psychological resilience, and also confirmed that good psychological resilience influences health. Therefore, this paper attempts to explore whether psychological resilience serves as a mediating variable in the influence of social network patterns on elderly health.

In summary, it can be seen that social network patterns, which better reflect the complexity of interpersonal environments [13], have become a popular research topic in recent years. Existing literature has confirmed the impact of social network patterns on elderly health. **However, there are the following limitations.** First, current academic research in social network analysis primarily focuses on single aspects of social network relationships, with relatively few studies on social network patterns. Second, research based on China’s national conditions is currently limited, and existing research data all come from specific regions, calling for more complete, comprehensive, and in-depth studies. Third, existing research mostly focuses on direct health impacts, lacking discussion of the mediating mechanisms through which social network patterns influence elderly health.

Therefore, based on literature review, theoretical analysis and summary, this paper attempts to examine the classification of social network patterns among Chinese elderly and the impact of different social network patterns on elderly health using a more comprehensive Chinese database. Additionally, it explores the influence mechanism using psychological resilience as a bridge. **The paper proposes the following hypotheses**. H1:Different social network patterns have different impacts on elderly health. H2:Psychological resilience plays a mediating role between social network patterns and elderly health.

**The potential contributions of this paper are threefold.** First, regarding perspective, starting from social network patterns and centering on the elderly, this study comprehensively examines social network characteristics from a holistic perspective, partially addressing the research limitations caused by social network complexity, and enriching research on social network patterns and elderly health in China. Second, empirically, based on a national micro-database, this study uses cluster analysis to calculate elderly social network patterns, quantifies the impact of different social network patterns on health, and reveals the influence pathway of psychological resilience between social network patterns and elderly health, which helps discover general patterns in how social network patterns affect elderly health. Third, in terms of practical significance, given the interventional nature of social network patterns and psychological resilience, the findings of this study have important implications for developing effective health interventions to improve elderly health conditions and quality of life, and promote healthy aging.

## 2. Data and methods

### 2.1 Data sources

This study uses cross-sectional data from the Chinese Longitudinal Healthy Influence Factor Tracking Survey (CLHLS) tracking data in 2018. The survey project was jointly organized by Peking University and Duke University, and covered information on family structure, economic status, and health of older adults. The survey program conducted a baseline survey in 1998, followed by follow-up surveys in 2000, 2002, 2005, 2008–2009, 2011–2012, 2014, and 2017–2018. The survey used a multi-stage stratified sampling method to randomly select half of the counties, cities or municipal districts in China’s representative 22 provinces, municipalities and autonomous regions for household interviews, and the population of the surveyed areas accounted for 85.3% of the country’s total population.The wealth of information contained in the CLHLS database facilitates the comprehensive analysis and study of the impact of social network types on the health of older adults.

In conjunction with the purpose of the study and data availability, this study used the CLHLS 2018 cross-sectional data, selecting older adults aged 60 years and older from the database, and deleting samples with missing values in the study variables, resulting in a final sample size of 8,042 individuals.

### 2.2 Selection of indicators

**Dependent variables**. The outcome variable in this study is health, which contains two dimensions of objective health and subjective health, and the presence of chronic diseases and self-assessed health are chosen as proxy variables, respectively. According to the questionnaire “whether there are chronic diseases”, those who choose to have the following hypertension, diabetes, heart disease, stroke and cerebrovascular disease bronchitis, asthma or pneumonia, tuberculosis, cancer are regarded as 1, and those who don’t are regarded as 0. Self-assessment of health in the questionnaire title is “Do you think that now your health status? How is it?” In this study, drawing on [35], the questionnaire defines very healthy, healthy, and relatively healthy as good self-assessed health and assigns a value of 1, while average and unhealthy are defined as poor self-assessed health and assigned a value of 0. Therefore, both chronic diseases and self-rated health are dichotomous variables. This facilitates analytical methods such as logistic regression, allowing for more intuitive study of the marginal effects of various factors on health outcomes.

**Independent variables.** The independent variables in this study are social network types, which are obtained by social network relationship size, contact frequency and functional support through cluster analysis. Using the k-mean cluster analysis method, four social network types, limited, family, friend and diverse, were obtained and assigned the values of limited-type social network pattern = 1, family-type social network pattern = 2, friend-type social network pattern = 3 and diverse-type social network pattern = 4, respectively, and the operationalization method is detailed in 2.3.

**Fig 1:**
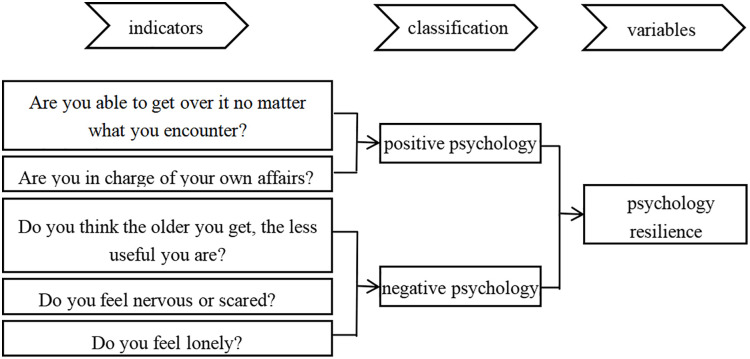
Psychological resilience.

**Mediating variable.** The mediating variable in this study is psychological resilience, including positive and negative psychology. this study draws on Liu Yujiang’s approach [6], choosing the questionnaire’s questions “Are you able to get over it no matter what you encounter?”, “Are you in charge of your own affairs?” as the positive psychology, “Do you think the older you get, the less useful you are?” “Do you feel nervous or scared?” “Do you feel lonely?” for negative psychology. All five questions are on a 5-point Likert scale, in which the two questions of positive psychology are reverse questions, and this study assigns reverse scores, and then all the scores of the questions are summed up to get the psychological resilience variable, which ranges from 5–25 points, see Fig 1 for details.

**Control variables.** Since the health of the elderly is affected by many factors, this study categorizes the control variables into four major aspects: demographic characteristics, socioeconomic factors, lifestyle and healthcare based on research needs and data availability. Demographic factors are gender, age, and current place of residence; and socioeconomic factors are education level and income level.In 1992, the World Health Organization (WHO) first proposed the concept of the four cornerstones of health in the famous Victoria Declaration, which are reasonable diet, moderate exercise, abstinence from smoking and alcohol, and psychological balance [36]. Thus, lifestyle includes whether one eats fresh vegetables regularly, eats meat regularly, exercises regularly, smokes now, and drinks alcohol regularly. Healthcare factors consider health insurance, and the type of health insurance is categorized as none = 0, public healthcare = 1, urban workers/residents health insurance = 2, new rural cooperative health insurance = 3, commercial health insurance = 4, and other = 5, as shown in Table 1.

**Table 1:** Variable assignment and selection.

Variables	Categorization	Variable name	Variable assignment
Dependent variable	Health	Objective health	With chronic disease = 1; without chronic disease = 0
		Self-assessment of health	Very healthy, very healthy, relatively healthy = 1; Fair, unhealthy = 0
Independent variable	Social network types	Social network types	Cluster analysis yielded that four types limited-type = 1, family-type = 2, friend-type = 3, and Multi-type = 4
Control variable	Demography	Gender	Male = 1; Female = 0
		Age	60 and over
		Current address	Urban = 1; Rural = 0
	Socio-economic	Educational attainment	Continuous variables, 0–14 points
		Income level	Enough income = 1; not enough income = 0
	Lifestyle	Vegetable intake	Regularly eat vegetables = 1; infrequently eat vegetables = 0
		Meat intake	Eat meat regularly = 1; eat meat infrequently = 0
		Physical exercise	Regular exercise = 1; infrequent exercise = 0
		Cigarette smoking	Smoking = 1; no smoking = 0
		Drink (alcohol)	Drinking = 1; not drinking = 0
	Health care	Medical insurance	None = 0, public medical care = 1, urban workers/residents’ medical insurance = 2, new rural cooperative medical insurance = 3, commercial medical insurance = 4, other = 5
Intermediary variable	Psychological resilience	Psychological resilience	Continuous variables, 5–25 points

The objective factual statistics for each variable are detailed in Table 2.

**Table 2:** Factual statistics on objectivity of variables.

Variables	Obs	Mean/N(%)	Std. Dev.	Min	Max
Chronic disease	8042	0.235	0.424	0	1
Self-assessed health	8042	0.867	0.34	0	1
Social network types	8042	2.068	1.046	1	4
Limited-type		2,945(36.63%)			
Family-type		2,801 (34.82%)			
Friend-type		1,097(13.64%)			
Diverse-type		1,199(14.91%)			
Psychological resilience	8042	19.436	3.012	6	25
Gender	8042	0.464	0.499	0	1
Age	8042	82.55	10.754	65	105
Educational attainment	8042	4.379	4.269	0	14
Current address	8042	0.542	0.498	0	1
Income level	8042	0.865	0.341	0	1
Vegetable intake	8042	0.909	0.287	0	1
Meat intake	8042	0.213	0.409	0	1
Physical exercise	8042	0.326	0.469	0	1
Cigarette smoking	8042	0.162	0.369	0	1
Drink (alcohol)	8042	0.163	0.369	0	1
Medical insurance	8042	2.410	1.079	0	5
None		997(12.4%)			
Public medical care		172(2.14%)			
Urban workers/residents		1787(22.22%)			
New rural cooperative		4873(60.59%)			
Commercial insurance		46(0.57%)			
Other		167(2.08%)			

### 2.3 Calculation of k-mean clustering for social network types

**Fig 2:**
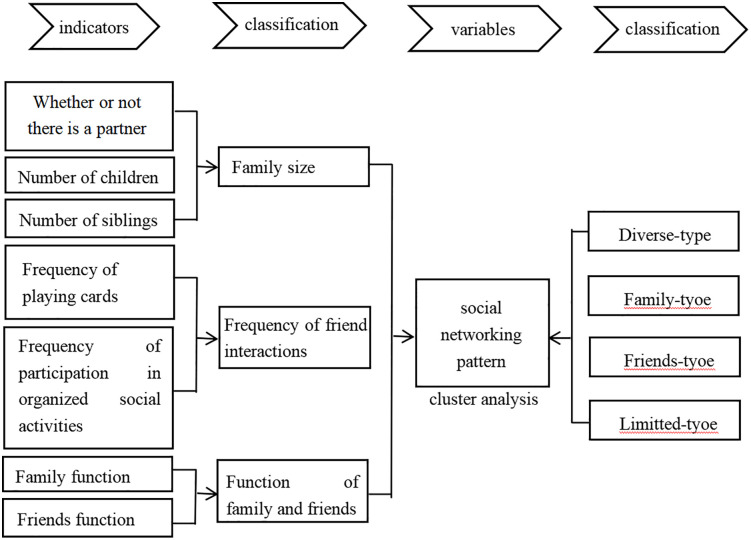
Social network model.

Social network types are the result of categorizing social networks according to their size, frequency of contact, and functionally supported characteristics. Among the components that make up the social network model, network size includes the presence or absence of a partner, which includes both the situation where a spouse is still alive after marriage and the situation where one is not married but has a partner, with the presence of a partner being scored as 1 and the absence of a partner being scored as 0. The network size also includes the number of close children and the number of siblings, both of which are continuous variables. Network contact frequency is mainly the frequency of friends’ network contact, including the frequency of playing cards and mahjong with friends, the frequency of participating in organized social activities, and the frequency of interaction is scored from 0–4 from low to high. Network function support support draws on scholar Li Ting’s treatment [22], based on the CLHLS questionnaire’s “Who do you chat with most on a daily basis??”, “If you have something on your mind or thoughts, who do you tell first?”, “Who is the first person you want to talk to if you have a problem or issue?” Three questions were sought. The options for the three questions were set up with 10 answers, all members of the social network, with options 0–6 being family members, 7 being friends, 8 being social workers, 9 being babysitters, and 10 being no one to chat with. We recorded older adults who chose options 0–6 as receiving functional support from family, 7 as receiving functional support from friends, and options 8–10 were not considered. Meanwhile, the scoring methodology for functional support employs a sophisticated weighting system that reflects both the importance and accessibility of different types of support relationships. For the daily chat question, respondents can select up to three network members, with responses weighted as follows: the first choice receives 3 points, the second choice 2 points, and the third choice 1 point. For the questions addressing personal concerns and problem-solving, respondents can select up to two network members each, with the first choice weighted at 2 points and the second choice at 1 point. Through this calculation method, family functional support scores range from 0 to 12 points, while friend functional support scores span from 0 to 7 points. See Fig 2 for details.

In order to get the social network types, this study draws on the processing method of [37] scholars to classify the social network contents using the k-mean clustering method. k-mean clustering method is a widely used clustering computation method, with the advantage of simplicity and efficiency. The basic idea is to take the normalized data as the basis, randomly select k centers among n data, divide the data in the sample into the nearest centroid categories, form k initial data sets, and then recalculate the clustering centers for each set, and iterate until the clustering centers no longer change.

The basic idea of k-mean clustering contains its specific steps:

(1) Randomly select k samples as initial cluster centers $\text{C}=\left\{c_{\text{1},\;}c_{\text{2},\;}\ldots c_{k\;}\right\}$.
(2) For each sample $x_{i}$ in the data, the distance from the sample to the center of the cluster is calculated separately and it is classified into the nearest centroid category.
(3) For each category $c_{j}$, recalculate the clustering center $c_{j}^{\prime }=\frac{\text{1}}{n}\sum_{x_{i\in }c_{j}}x_{i}$, where n is the category and $c_{j}$ is the number of data points.

Repeat steps (2) and (3) until the center of clustering no longer changes.

In this study, seven variables, namely whether there is a partner, number of children, number of siblings, frequency of playing cards or mahjong, frequency of participating in organized social activities, family function and friend function, were used for k-mean clustering, and a total of 32 iterations were used to identify four social network types, which were classified as diverse, family, friends and limited.

To systematically demonstrate the empirical process of k-means clustering analysis, the following sequentially presents the key steps in the analysis. The empirical application of this framework draws upon comprehensive measurements across 8,042 older adults, providing robust statistical foundation for the clustering analysis. Table 3 presents the descriptive characteristics of all seven clustering variables.

**Table 3:** Descriptive statistics for seven clustering variables.

Variable	Obs	Mean	Std. Dev.	Min	Max
Partner presence	8,042	0.492	0.500	0	1
Number of close children	8,042	3.558	1.668	0	11
Number of siblings	8,042	1.735	1.709	0	10
Frequency of playing cards/mahjong	8,042	0.528	1.200	0	4
Frequency of social activities	8,042	0.337	0.907	0	4
Family functional support	8,042	9.325	2.761	0	12
Friend functional support	8,042	0.736	1.322	0	7

Prior to conducting the clustering analysis, it is essential to determine the optimal number of clusters. Determining the optimal number of clusters requires balancing statistical optimization with theoretical interpretability and practical utility. Our systematic evaluation process examined solutions ranging from 2 to 6 clusters using multiple criteria including within-cluster sum of squares (WCSS), Calinski-Harabasz index, elbow method assessment, and theoretical interpretability. The comprehensive comparison of these criteria is presented in Table 4. The statistical analysis reveals that k = 4 provides the most compelling combination of statistical adequacy and theoretical coherence. The four-cluster solution demonstrates good performance across all metrics: a strong Calinski-Harabasz index of 1711.77 and a WCSS of 16,044.30, and most importantly, the resulting typology aligns with Litwin’s internationally validated framework while adapting to the specific characteristics of Chinese older adults, creating distinct, meaningful network types that maintain both statistical rigor and theoretical grounding. This solution creates distinct, meaningful network types that align with established social network theory while maintaining sufficient statistical rigor.

**Table 4:** K-selection analysis with multiple evaluation criteria.

	Calinski-Harabasz index	WCSS
k = 2	1607.79	19,327.90
k = 3	1564.96	17,315.30
k = 4	1711.77	16,044.30
k = 5	1025.95	13,951.50
k = 6	1621.06	12,918.10

Having established k = 4 as the optimal cluster solution, we proceeded to analyze the specific characteristics of each cluster. The resulting four-cluster typology reveals distinct and theoretically coherent social network patterns that demonstrate clear differentiation across all analytical dimensions. Table 5 presents the detailed cluster profiles, showing significant between-group differences (all p < 0.001) with substantial effect sizes ranging from η² = 0.049 to η² = 0.784, indicating that the clustering variables effectively distinguish between network types.

**Table 5:** Four-cluster profiles across seven variables.

Variable	Limited-type	Family-type	Friend-type	Diverse-type	F-statistic	p-value	η²
**Partner presence**	0.018 (0.134)	0.998 (0.050)	0.176 (0.381)	0.765 (0.424)	9709.18	<0.001	0.784***
**Number of close children**	3.975 (1.762)	3.414 (1.548)	3.529 (1.604)	2.895 (1.475)	138.46	<0.001	0.049***
**Number of siblings**	1.103 (1.398)	2.146 (1.749)	1.714 (1.692)	2.344 (1.811)	262.36	<0.001	0.089***
**Frequency of playing cards/mahjong**	0.108 (0.505)	0.103 (0.372)	0.479 (1.149)	2.597 (1.538)	3048.54	<0.001	0.532***
**Frequency of social activities**	0.082 (0.376)	0.142 (0.422)	0.279 (0.819)	1.472 (1.612)	1038.09	<0.001	0.279***
**Family functional support**	9.593 (2.898)	10.180 (2.013)	5.836 (2.659)	9.862 (2.157)	3662.58	<0.001	0.577***
**Friend functional support**	0.198 (0.462)	0.324 (0.698)	3.467 (1.145)	0.524 (0.939)	5712.82	<0.001	0.681***
**N**	2,946	2,800	1,097	1,199			
**%**	36.63%	34.82%	13.64%	14.91%			

Note: Values are means with standard deviations in parentheses. ***p < 0.001. η² represents effect size.

The cluster size distribution reveals that Limited-type networks constitute the largest group (36.63%, n = 2,946), followed closely by Family-type (34.82%, n = 2,800), while Friend-type (14.91%, n = 1,199) and Diverse-type (13.64%, n = 1,097) represent smaller but distinct segments of the older adult population. The table reports cluster means and standard deviations for each variable, along with F-statistics, p-values, and effect sizes (η²) that quantify the statistical significance and practical importance of between-cluster differences.

Based on the cluster profiles, each network type exhibits distinct characteristics that reflect different social relationship strategies among older adults:

Limited-type networks (36.63%, n = 2,946) represent the most socially isolated pattern, characterized by very low partner presence (1.8%), moderate family size (3.98 children), but relatively low family functional support (9.59) and minimal friend engagement across all dimensions. This pattern suggests structural social connections that are not functionally activated, indicating potential social vulnerability.

Family-type networks (34.82%, n = 2,800) demonstrate the highest family orientation, with near-universal partner presence (99.8%), the strongest family functional support (10.18), and moderate family size (3.41 children). However, they show limited friend interactions and minimal functional support from friends (0.32), representing a family-centered social strategy that prioritizes kinship relationships.

Friend-type networks (13.64%, n = 1,097) show the opposite pattern with low partner presence (17.6%) but the highest friend functional support (3.47) and moderate friend interaction frequencies. Family functional support is notably lower (5.84) compared to family-oriented types, indicating a social strategy that compensates for limited family resources through friend-based relationships.

Diverse-type networks (14.91%, n = 1,199) represent the most socially integrated pattern, with high partner presence (76.5%), the highest frequencies of social activities (1.47) and card/mahjong playing (2.60), along with substantial functional support from both family (9.86) and friends (0.52). This type demonstrates successful integration across multiple relationship domains.

This typological framework provides a comprehensive understanding of how older adults structure their social relationships, revealing that social network organization follows distinct patterns rather than random variation. The four types capture the primary ways older adults navigate social connection: through comprehensive integration across multiple relationship types, through family-centered approaches, through friend-focused strategies, or through minimal social engagement. These patterns have significant implications for understanding social support, health outcomes, and intervention strategies for older adult populations, as each network type likely requires different approaches to maintaining social connection and well-being.

## 3. Results

### 3.1 Descriptive analysis

**Fig 3:**
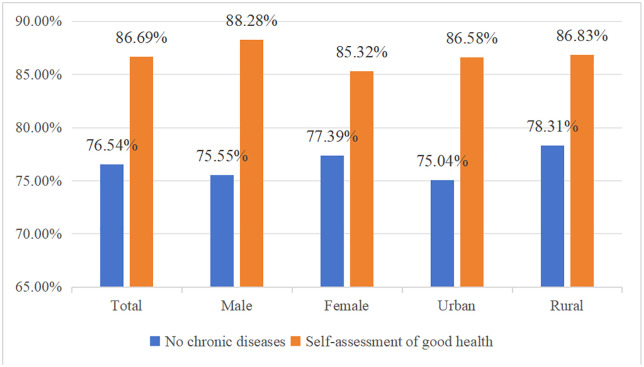
Health of the elderly.

**Fig 4:**
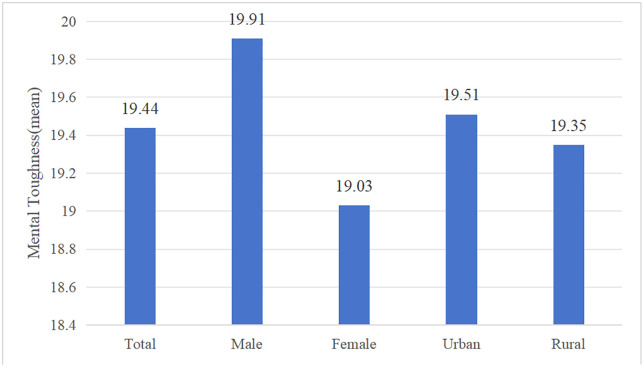
Psychological resilience status.

This study uses CLHLS 2018 microdata on the status of social network types, older adults’ health, and psychological resilience. The descriptive statistics are shown in Table 6, Figs 3 and 4. The social network model is dominated by limited and family type, the overall health of the elderly is better, and the average score of psychological resilience is 19.44. At the same time, there are some correlations between health of the elderly and social network types, social network types and psychological resilience and health of the elderly and psychological resilience. According to statistical data, older people with diverse-type social network have a higher probability of self-assessing their health status and not having chronic diseases, as well as a higher average score for psychological resilience; older people with good psychological resilience have a higher probability of better self-assessed health and not having chronic diseases.

**Table 6:** Status of social network types.

Social network types	Percentage of total sample	Male/Female		Urban/Rural	
		Male	Female	Urban	Rural
Diverse-type	14.91%	58.63%	41.37%	64.22%	35.78%
Family-oriented	34.82%	60.57%	39.43%	52.54%	47.46%
Friends-type	13.64%	37.92%	62.08%	50.96%	49.04%
Limitied-type	36.63%	68.94%	31.06%	52.92%	47.08%

### 3.2 Multicollinearity analysis

Multicollinearity was tested using variance inflation factors (VIFs). As shown in Table 7, the VIFs ranged from 1.03 to 2.46, with a mean VIF of 1.37, well below the conventional threshold of 5, indicating that multicollinearity was not a concern in our analysis.

**Table 7:** Results of multicollinearity diagnostics using Variance Inflation Factors (VIF).

Variable	VIF	1/VIF
Social night types		
2 = Family-oriented	1.88	0.5310
3 = Friends-type	1.32	0.7558
4 = Diverse-type	1.61	0.6207
Gender	1.32	0.7596
Age	1.54	0.6500
Current address	1.19	0.8371
Educational attainment	1.14	0.8768
Income level	1.04	0.9661
Vegetable intake	1.03	0.9735
Meat intake	1.09	0.9204
Physical exercise	1.12	0.8950
Cigarette smoking	1.21	0.8252
Drink (alcohol)	1.16	0.8595
Medical insurance		
1 = Public medical care	1.16	0.8593
2 = Urban workers/residents’ medical insurance	2.24	0.4473
3 = New rural cooperative medical insurance	2.46	0.4066
4 = Commercial medical insurance	1.04	0.9584
5 = Other	1.15	0.8709
Mean VIF	1.37	

### 3.3 Empirical results

Social network types are associated with better health outcomes have an effect on self-assessed health of older adults and a non-significant effect on the presence of chronic diseases. In the empirical research part, this study uses a binary Logit regression model to process and analyze the micro data. As the limited-type social network pattern characterized by relatively restricted social connections represents the most basic state of social relationships, choosing the Limited social network pattern as the reference group allows for a clear comparison of the effects brought about by different social network patterns. The marginal effects of the model variables on the health of the elderly are shown in Table 8. In order to further explore the influence of social network model on the health of the elderly, this study adopts gender and urban-rural heterogeneity for the study, and the specific empirical results are detailed in Tables 9 and 10. To explore whether psychological resilience serves as a mediating mechanism for the social network model to influence the self-assessed health of the elderly, this study firstly treats the social network model as an integral whole, and drawing on the mediating effect model of [38], observing the connection between social network types and psychological resilience and self-assessed health, and determining whether its mediating mechanism exists, as shown in Tables 11. The social network types were then refined and categorized to test the mechanisms by which different social network types affect older adults’ self-rated health through the mediating factor of psychological resilience.

**Table 10:** Impact of social network types on the health of older adults: an analysis of urban-rural based differences.

Explanatory variable	Objective health (chronic diseases)		Subjective health (self-rated health)	
	Logit marginal utility			
	Urban	Rural	Urban	Rural
Social network model (with limited type as reference group)				
Family-oriented	−0.0045	0.0250	−0.0190	−0.0127
	(0.0189)	(0.0189)	(0.0147)	(0.0157)
Friends-type	−0.0121	0.0459^**^	−0.0005	−0.0176
	(0.0224)	(0.0222)	(0.0172)	(0.0178)
Diverse-type	−0.0063	0.0017	0.0077	0.0521^***^
	(0.0223)	(0.0250)	(0.0170)	(0.0180)
Gender	0.0317^**^	0.0303^*^	0.0143	0.0165
	(0.0146)	(0.0155)	(0.0114)	(0.0128)
Age	0.0005	−0.0008	0.0005	0.0005
	(0.0008)	(0.0008)	(0.0006)	(0.0006)
Educational attainment	−0.0018	0.0002	−0.0026^**^	0.0044^***^
	(0.0015)	(0.0018)	(0.0012)	(0.0016)
Income level	−0.0768^***^	−0.0762^***^	0.1284^***^	0.1101^***^
	(0.0199)	(0.0170)	(0.0128)	(0.0121)
Vegetable intake	−0.0157	−0.0342	0.0527^***^	0.0407^**^
	(0.0224)	(0.0228)	(0.0152)	(0.0169)
Meat intake	0.0138	−0.0148	0.0111	−0.0157
	(0.0149)	(0.0200)	(0.0120)	(0.0159)
Physical exercise	−0.0086	0.0281^*^	0.0893^***^	0.0428^***^
	(0.0143)	(0.0156)	(0.0122)	(0.0141)
Cigarette smoking	0.0220	0.0405^*^	0.0103	0.0052
	(0.0199)	(0.0207)	(0.0163)	(0.0172)
Drink (alcohol)	0.0653^***^	0.0635^***^	0.0807^***^	0.0519^***^
	(0.0199)	(0.0211)	(0.0184)	(0.0187)
Health insurance (with no health insurance as the reference group)				
Medical treatment at public expense	0.0478	0.0237	0.0816^**^	0.0140
	(0.0429)	(0.0704)	(0.0390)	(0.0604)
Employee/Household Insurance	0.0526^**^	0.0133	0.0405^**^	0.0541^**^
	(0.0206)	(0.0322)	(0.0161)	(0.0273)
New Agricultural Cooperative Society (NACS)	−0.0337^*^	0.0271	0.0057	0.0224
	(0.0194)	(0.0227)	(0.0146)	(0.0202)
Commercial insurance	0.1711	−0.0240	0.0000	0.1147^***^
	(0.1049)	(0.0791)	(0.0000)	(0.0430)
Other medical insurance	0.0262	0.1879^***^	0.0198	0.0014
	(0.0474)	(0.0616)	(0.0325)	(0.0446)
Sample size	4359	3683	4359	3683
Pseudo R2	0.0136	0.0175	0.0671	0.0558

**Table 11:** Mechanistic analysis of the impact of social network types on self-rated health of older adults.

Variable	Subjective health (self-assessed health)	Psychological resilience	Subjective health (self-assessed health)
	Logit model		
Social network types	0.0554^*^	0.2301^***^	0.0016
	(0.0332)	(0.0339)	(0.0387)
Psychological resilience			0.2571^***^
			(0.0125)
Gender	0.1840^**^	0.5148^***^	−0.0362
	(0.0735)	(0.0708)	(0.0787)
Age	0.0028	−0.0180^***^	0.0113^***^
	(0.0021)	(0.0033)	(0.0036)
Educational attainment	0.0015	0.0162^**^	−0.0009
	(0.0082)	(0.0078)	(0.0086)
Current address	−0.0900	−0.1912^***^	−0.0346
	(0.0727)	(0.0691)	(0.0761)
Income level	1.1203^***^	1.5071^***^	0.7859^***^
	(0.0802)	(0.0938)	(0.0863)
Vegetable intake	0.4330^***^	0.9132^***^	0.2094^*^
	(0.1030)	(0.1103)	(0.1085)
Meat intake	0.0096	0.0801	−0.0275
	(0.0868)	(0.0802)	(0.0907)
Physical exercise	0.6531^***^	0.8821^***^	0.4369^***^
	(0.0833)	(0.0710)	(0.0871)
Cigarette smoking	0.2006^*^	0.1249	0.0122
	(0.1051)	(0.0941)	(0.1118)
Drink (alcohol)	0.6335^***^	0.5217^***^	0.5467^***^
	(0.1196)	(0.0920)	(0.1239)
Health insurance (with no health insurance as the reference group)			
Medical treatment at public expense	0.3642^*^	0.0460	0.4810^*^
	(0.2044)	(0.0362)	(0.2510)
Employee/Household Insurance	0.1597	0.0532^***^	0.2721
	(0.1949)	(0.0171)	(0.2399)
New Agricultural Cooperative Society (NACS)	0.4570^**^	−0.0055	0.6455^***^
	(0.1831)	(0.0147)	(0.2366)
Commercial insurance	2.3767^**^	0.0672	2.4931^**^
	(1.0304)	(0.0684)	(1.0580)
Other medical insurance	0.4317	0.0921^**^	0.4978
	(0.2967)	(0.0379)	(0.3351)
Intercept term	0.1196	17.9231^***^	−4.8522^***^
	(0.3699)	(0.3576)	(0.4431)
Sample size	8042	8042	8042
Pseudo R2	0.0547	0.1214	0.1285

Note: It is not necessary to analyze the economic significance of specific coefficients in the mechanism analysis, so the above table is for logit coefficients, not marginal utility.

**Table 8:** Results of logit regression marginal effects of social network types on the health of older adults.

Variables	Objective health (chronic diseases)		Subjective health (self-rated health)	
	Logit marginal utility			
Social network model (with limited type as reference group)				
Family-oriented	0.0161	0.0099	−0.0064	−0.0161
	(0.0112)	(0.0135)	(0.0092)	(0.0108)
Friends-type	0.0213	0.0166	−0.0063	−0.0103
	(0.0151)	(0.0159)	(0.0123)	(0.0124)
Diverse-type	0.0028	−0.0017	0.0459^***^	0.0232^*^
	(0.0144)	(0.0165)	(0.0104)	(0.0126)
Gender		0.0321^***^		0.0149^*^
		(0.0107)		(0.0085)
Age		−0.0000		0.0004
		(0.0005)		(0.0004)
Educational attainment		−0.0007		−0.0003
		(0.0012)		(0.0009)
Current address		0.0171^*^		−0.0095
		(0.0104)		(0.0081)
Income level		−0.0777^***^		0.1189^***^
		(0.0131)		(0.0088)
Vegetable intake		−0.0272^*^		0.0487^***^
		(0.0161)		(0.0114)
Meat intake		0.0066		−0.0000
		(0.0118)		(0.0095)
Physical exercise		0.0069		0.0701^***^
		(0.0105)		(0.0093)
Cigarette smoking		0.0308^**^		0.0085
		(0.0144)		(0.0119)
Drink (alcohol)		0.0644^***^		0.0679^***^
		(0.0145)		(0.0131)
Health insurance (with no health insurance as the reference group)				
Medical treatment at public expense		0.0460		0.0586^*^
		(0.0362)		(0.0336)
Employee/Household Insurance		0.0532^***^		0.0240^*^
		(0.0171)		(0.0142)
New Agricultural Cooperative Society (NACS)		−0.0055		0.0095
		(0.0147)		(0.0118)
Commercial insurance		0.0672		0.1120^***^
		(0.0684)		(0.0243)
Other medical insurance		0.0921^**^		0.0112
		(0.0379)		(0.0264)
Sample size	8042	8042	8042	8042
Pseudo R2	0.0004	0.0132	0.0038	0.0563

Note: (1) Robust standard errors in parentheses; (2) * denotes p < 0.1, ** denotes p < 0.05, and *** denotes p < 0.01; (3) logit regression marginal utility does not show a constant term; same below.

**Table 9:** Impact of social network types on the health of older adults: analysis of differences based on gender.

Explanatory variable	Objective health (chronic diseases)		Subjective health (self-rated health)	
	Logit marginal utility			
	Male	Female	Male	Female
Social network model (with limited type as reference group)				
Family-oriented	0.0388^*^	−0.0243	−0.0227	−0.0141
	(0.0199)	(0.0181)	(0.0147)	(0.0158)
Friends-type	−0.0007	0.0242	−0.0099	−0.0117
	(0.0254)	(0.0202)	(0.0193)	(0.0165)
Diverse-type	0.0292	−0.0319	0.0080	0.0359^**^
	(0.0243)	(0.0222)	(0.0173)	(0.0182)
Age	0.0004	−0.0006	0.0006	0.0002
	(0.0008)	(0.0007)	(0.0006)	(0.0006)
Educational attainment	−0.0030	0.0014	−0.0014	0.0005
	(0.0018)	(0.0015)	(0.0013)	(0.0013)
Current address	0.0220	0.0135	−0.0033	−0.0144
	(0.0154)	(0.0140)	(0.0113)	(0.0115)
Income level	−0.0380^*^	−0.1063^***^	0.1116^***^	0.1261^***^
	(0.0204)	(0.0169)	(0.0124)	(0.0126)
Vegetable intake	−0.0462^*^	−0.0093	0.0456^***^	0.0523^***^
	(0.0244)	(0.0211)	(0.0163)	(0.0158)
Meat intake	−0.0016	0.0163	−0.0052	0.0069
	(0.0168)	(0.0165)	(0.0124)	(0.0144)
Physical exercise	−0.0072	0.0204	0.0689^***^	0.0680^***^
	(0.0151)	(0.0147)	(0.0122)	(0.0137)
Cigarette smoking	0.0433^***^	0.0222	0.0093	0.0009
	(0.0164)	(0.0315)	(0.0121)	(0.0278)
Drink (alcohol)	0.0788^***^	−0.0285	0.0757^***^	0.0367
	(0.0171)	(0.0280)	(0.0143)	(0.0250)
Health insurance (with no health insurance as the reference group)				
Medical treatment at public expense	0.0614	−0.0006	0.0460	0.0973
	(0.0444)	(0.0641)	(0.0364)	(0.0687)
Employee/Household Insurance	0.0606^**^	0.0406^*^	0.0321^*^	−0.0145
	(0.0250)	(0.0234)	(0.0192)	(0.0205)
New Agricultural Cooperative Society (NACS)	0.0018	−0.0117	0.0032	0.0145
	(0.0223)	(0.0196)	(0.0165)	(0.0168)
Commercial insurance	−0.0017	0.1100	0.0000	0.1151^***^
	(0.0990)	(0.0921)	(0.0000)	(0.0405)
Other medical insurance	0.0625	0.1134^**^	0.0329	−0.0036
	(0.0578)	(0.0500)	(0.0340)	(0.0384)
Sample size	3730	4312	3730	4312
Pseudo R2	0.0176	0.0154	0.0684	0.0451

The results support Hypothesis 2. According to the results of the empirical analysis, the probability of self-assessed health being “better” was 4.59% higher in the diverse social network model than in the limited social network model. There was no significant effect of the social network model of friends and family on the self-rated health of older adults. There was no significant effect of the social network model on objective health (chronic diseases) among older adults.

There is **gender and urban-rural heterogeneity in the** impact of social network types on the health of older adults**, as detailed in Tables 9 and 10**.The regression results show that male older adults have a higher probability of having a chronic disease in the family-type social network pattern than in the limited-type, and that female older adults have a non-significant effect of the social network pattern on the presence or absence of a chronic disease. Diverse-type social network pattern had better self-assessed health than limited type for female older adults, and social network pattern of male older adults had insignificant effect on self-assessed health. Rural older adults’ friend-based social network pattern has a higher probability of having a chronic disease than the limited type, and urban older adults’ social network pattern has a non-significant effect on the presence of a chronic disease. Rural older adults with diverse social network types had better self-assessed health than limited patterns, and urban older adults’ social network types had a non-significant effect on self-assessed health.

**Fig 5:**
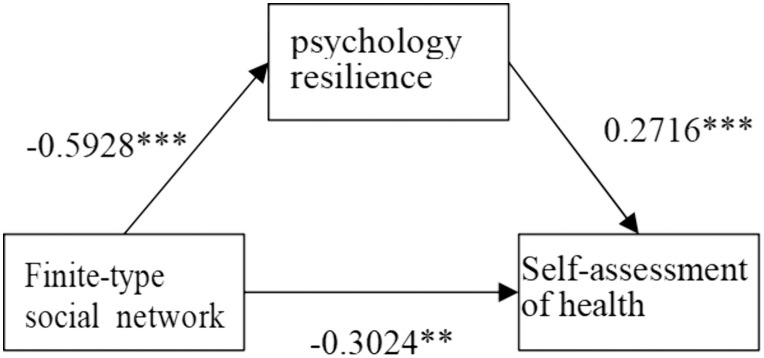
Mechanisms of limited-type influence on self-rated health.

**Fig 6:**
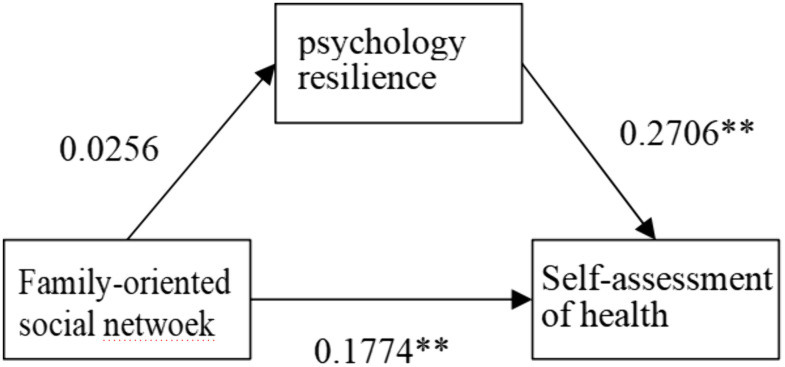
Mechanisms of family-type influence on self-rated health.

**Fig 7:**
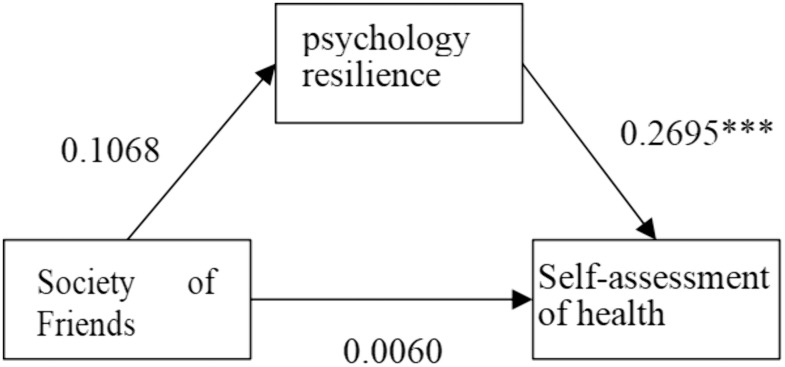
Mechanisms of friendship-type’s impact on self-rated health.

**Fig 8:**
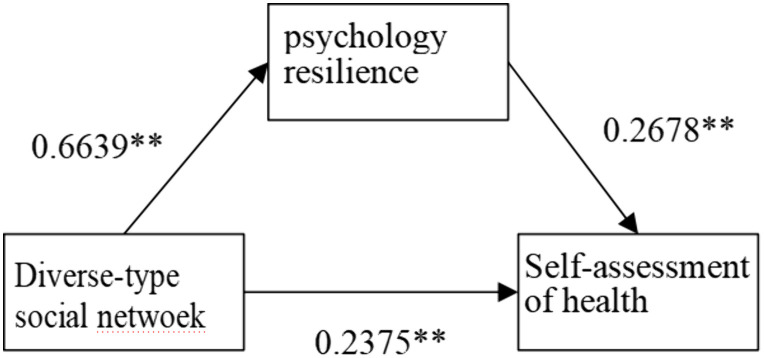
Mechanisms of diverse- type’s impact on self-rated health.

**As shown in** Table 11 **and** Figs 5–8, psychological resilience **has a mediating mechanism** in the effects of social network types on older adults’ self-assessed health. The results support Hypothesis 3.

Overall, the research finds that social network patterns affect elderly people’s subjective health status through psychological resilience, demonstrating a complete mediating effect of psychological resilience. This complete mediation is evident when controlling for psychological resilience, as the direct effect of social networks on subjective health becomes insignificant, while the impact of psychological resilience on health remains significant.

However, when examining different types of social networks separately, more complex influence mechanisms emerge. Psychological resilience plays a partial mediating effect in both limited-type and diverse-type social network types, with the former negatively affecting older adults’ self-assessed health through psychological resilience and the latter positively affecting older adults’ self-assessed health through psychological resilience, that is, the limited type of social network model decreases the level of psychological resilience of the elderly and thus worsens older adults’ self-assessed health, while the opposite is true for the diverse type. The family social network model has a direct positive effect on older adults’ self-rated health. The Friends model had no significant effect on older adults’ self-rated health. This differentiated pattern of influence indicates that while social networks generally affect elderly health primarily through enhancing psychological resilience, different types of social networks vary significantly in their pathways and strength of influence, providing important basis for developing targeted health intervention strategies for the elderly.

### 3.4 Robustness tests

There are many factors affecting the health of the elderly, and endogeneity problems arising from the omission of key variables often occur in related studies. Robustness tests are conducted to exclude the effect of endogenous variables on the regression results. In this study, in order to test the robustness of the regression results, fixed-effects regression analysis is conducted using the Chinese Longitudinal Healthy Influence Factor Tracking Survey (CLHLS) tracking data for the three periods of 2011–2012, 2014, and 2017–2018, which constitute panel data. The advantages of using fixed-effects regression analysis are (1) fixed effects can yield credible unbiased estimated coefficients, (2) genetics is often an important factor in health in related studies, and fixed-effects models can control for some individual characteristics that do not change over time, such as genes. The results of the specific robustness tests are shown in Table 12.

**Table 12:** Results of robustness tests on the effect of social network types on psychological resilience and self-rated health (three periods of panel data).

Explanatory variable	Objective health (chronic diseases)		Subjective health (self-rated health)	
	Xtlogit model			
Social network model (with limited type as reference group)				
Family-oriented	0.0897	0.0544	−0.0452^*^	−0.0388
	(0.0624)	(0.0762)	(0.0264)	(0.0277)
Friends-type	0.1181	0.0904	−0.0344	−0.0227
	(0.0827)	(0.0880)	(0.0268)	(0.0265)
Diverse-type	0.0159	−0.0105	0.1220^***^	0.1109^***^
	(0.0817)	(0.0951)	(0.0362)	(0.0357)
Gender		0.0282^***^		0.0034
		(0.0031)		(0.0038)
Current address		0.0964^*^		−0.0843
		(0.0585)		(0.0736)
Income level		−0.4364^***^		1.0857^***^
		(0.0743)		(0.0815)
Vegetable intake		0.1343		0.4387^***^
		(0.0904)		(0.1031)
Meat intake		0.0409		0.0017
		(0.0665)		(0.0869)
Physical exercise		0.1371^**^		0.6360^***^
		(0.0595)		(0.0842)
Cigarette smoking		0.1768^**^		0.0748
		(0.0813)		(0.1079)
Drink (alcohol)		0.0363		0.6236^***^
		(0.0820)		(0.1196)
Individual fixed effect	containment	containment	containment	containment
Time fixed effect	containment	containment	containment	containment
Sample size	3714	3714	3714	3714
Log likelihood	−4379.8496	−4324.5001	−3141.5436	−2976.4777

Note: The gender, years of education, and health insurance variables do not vary with survey time point in the sample, and the effects of these three are included in the individual fixed effects, so they are not included in this table.

**The results of the panel data regression analysis are basically consistent with the results of the benchmark regression.** According to Table 12, based on the panel data regression results, social network types have no significant effect on whether older adults suffer from chronic diseases, and diverse-type social network pattern has a significant positive effect on older adults’ self-assessed health, which is consistent with the results of the benchmark regression. As we can see from the table, the marginal effect of diverse social network types on the self-assessed health of older adults is significant at the 1% level, while other variables remain unchanged. Specifically, the probability of self-assessed health being “better” is 12.2% higher for diverse-type social network pattern than for limited-type social network pattern. After adding other control variables, the marginal effect of diverse-type social network model on older adults’ self-rated health is still significant at the 1% level, specifically, diverse-type social network model is 11.09% more likely than limited-type social network model to have “better” self-rated health. The probabilistic results for the panel data are greater than the baseline regression results because the tracking data partially control for the influence of genetic factors and better visualize the role of social network types.

## 4. Discussion

This study found that the social network types of Chinese older adults were categorized into four types: diverse-type, family-type, friend-type and limited-type after the clustering method. This categorization is consistent with common international categorization [14].

Overall, all of our two hypotheses received support. Consistent with Hypothesis 1, different social network patterns have varying effects on elderly people’s health. Among the social network models, the diverse-type social network model had a 4.59% higher probability of having “better” self-assessed health than the limited-type social network model. The friend-type and family-type social network models did not have a significant effect on the self-rated health of older adults. There was no significant effect of social network model on objective health (chronic diseases) of older adults. The results of this study are different from the results of existing international literature. Scholars in Western countries believe that the friend-type social network model plays a greater role [10], while Eastern scholars represented by Japan and South Korea believe that the family-type social network model has a greater impact on health [16]. However, the results of this study are consistent with the findings of existing studies by Chinese scholars, which suggest that people with diverse-type social network pattern have better health [19,9]. It can be inferred that perhaps the differences are brought about by the different cultures and customs of the country.

The results provided strong support for Hypothesis 2. This study found that psychological resilience has a mediating role in the effect of social network types on older adults’ self-rated health. This finding aligns with previous studies suggesting that social resources operate through psychological mechanisms to influence health outcomes [39,40]. This suggests that social network patterns enhance older adults’ psychological resilience, facilitating their adaptation to life circumstances and enabling more positive responses to life stressors and challenges, thereby maintaining better health status. Limited-type social network pattern reduces levels of psychological resilience and thus worsen older adults’ self-rated health, whereas the opposite is true for diverse-type social network pattern. These findings align with previous findings about the detrimental health impacts of social isolation [41] and research suggesting that diverse social ties provide complementary resources and support [42]. The mediating role of psychological resilience in the relationship between social network types and self-rated health among older adults may be attributed to the differences in social support and resources provided by various network types. Limited-type social networks offer fewer resources, making it difficult for older adults to develop the capacity to cope with stress and challenges, thus leading to decreased psychological resilience. In contrast, diverse-type social networks encompass rich social resources from different domains, enabling older adults to draw strength from them, enhance their psychological resilience, and consequently improve their self-rated health.

Furthermore, gender and urban-rural differences were observed in the impact of social network models on the health of older people. The social network model had a greater impact on the health of older women compared to older men. Similarly, the social network model had a greater influence on the health of older persons in rural areas than in urban areas. The observed gender and urban-rural disparities in the impact of social network models on older adults’ health may stem from the differences in gender roles and living environments. In traditional Chinese culture, women often bear more family responsibilities and emotional support roles, rendering the influence of social networks on their health more prominent. Rural areas have relatively simple and stable social networks, making it easier for older adults to obtain support, whereas urban elders face more complex and dynamic social environments, potentially diluting the impact of social networks due to the presence of other factors.

**This study makes theoretical and practical contributions to the field of geriatric health and social networks.**

**From a theoretical perspective**, this research enriches and deepens our understanding of the relationship between social network patterns and elderly health. First, by examining elderly health through the lens of social network patterns and employing convoy theory and cluster analysis, we systematically identified and classified all social network patterns radiating from elderly individuals as focal points. This quantitative analysis of different social network types’ impacts on elderly health transcends the limitations of existing research, which primarily focused on single dimensions of social networks. It provides a novel perspective for comprehensively understanding the health effects of social networks and enriches the literature on social network patterns and elderly health within the Chinese context. Second, this study reveals the mediating role of psychological resilience in how social network patterns influence elderly health, identifying the psychological pathway through which social networks affect health outcomes. This finding enriches the theoretical explanations of how social networks influence elderly health. Third, by comparing the differences in the relationship between social network patterns and elderly health across gender and urban-rural groups, this study helps clarify the heterogeneity in health effects of social network patterns, laying the foundation for constructing more comprehensive and nuanced theoretical models. In summary, this research theoretically integrates multiple analytical levels, including social network patterns, psychological resilience mechanisms, and demographic differences, contributing significantly to constructing a theoretical framework for health effects of social network patterns within the Chinese context.

**From the practical perspective**, this study provides crucial evidence for developing social network intervention strategies to promote elderly health. First, our finding that diverse social network patterns are most beneficial for elderly health suggests the importance of encouraging older adults to develop varied social connections and balance social interactions across different domains, including family and friends, to construct robust and rich support networks. Second, our verification of psychological resilience’s crucial role in how social network types promote elderly health implies the need to emphasize the cultivation of psychological resilience among older adults. By enhancing their confidence and skills in coping with stress and change, the health-promoting effects of social network patterns can be fully realized. Third, our identification of gender and urban-rural differences in how social network patterns affect elderly health indicates the necessity of considering group-specific characteristics and needs when developing intervention strategies. For instance, more opportunities for community participation could be provided to female and rural elderly residents to expand their social networks and offer additional care and support. In conclusion, this study provides practically targeted recommendations for government and social organizations to improve the social network environment and enhance health outcomes for the elderly population.

**Among the limitations of this study**, firstly, when analyzing the impact of social network model on the objective health of the elderly, this study selects the current data of social network model and the the objective health of the elderly to analyze the impact of social network model on the objective health of the elderly in the current situation. However, considering the possible delay and lag in the impact of social network model on the objective health of the elderly, it is necessary to analyze the impact of the current period social network model on the objective health of the elderly in the next period. However, it is also meaningful to explore the effect of social network model on subjective health of older adults using data from the current period, and if we want to study both subjective and objective health of older adults, the choice of data period will be contradictory, so this may require further thinking and learning, which is the direction that can be researched in the next period.

Secondly, the selection of control variable indicators is difficult because of the large number of factors affecting the health of the elderly. Although this study covers the four major aspects of demography, socio-economics, lifestyle and health care as much as possible, it is difficult to exhaust all the factors affecting the health of the elderly, so the selection of control variables in this study may be insufficient.

## 5. Conclusions

Summarizing the above empirical findings, it can be found that social network types have a significant impact on the health of older adults, with diverse-type social network pattern being significantly better than limited-type. psychological resilience has a mediating role in social network types and older adults’ health. There is a gender difference in the effect of social network types on the health of older adults, and social network types have a greater effect on the health of older adults in females compared to males. There are urban-rural differences in the impact of the social network model on the health of older people, with the social network model having a greater impact on the health of older people in rural areas than in urban areas.

Based on the latest relevant data, we calculated the social network types of Chinese older adults using cluster analysis method, explored the relationship and heterogeneity between social network types and older adults’ health through regression analysis, and analyzed the mediating role of psychological resilience through mechanisms analysis, which enriched the conclusions of the existing studies, and was helpful to promote the academic community’s attention to China’s social network types, to form a positive psychological awareness, to enhance the level of psychological resilience, and to promote the health improvement of the elderly.

Not only does it prompt older people to consciously open up wide to different types of people on a daily basis, encouraging older people to establish a variety of types of social networks, but it also reminds the Government to further establish and improve platforms that promote the enrichment of older people’s social networks and to establish personalized services and guidance for different groups of people. For example, the Government and communities should support and encourage older persons through the founding of the high-quality and diversified University of the Elderly, the provision of education for older persons and the development of activities for older persons to promote social interaction among older persons.
